# Policy issues related to educating the future Israeli medical workforce: an international perspective

**DOI:** 10.1186/s13584-015-0030-y

**Published:** 2015-10-01

**Authors:** Stephen C. Schoenbaum, Peter Crome, Raymond H. Curry, Elliot S. Gershon, Shimon M. Glick, David R. Katz, Ora Paltiel, Jo Shapiro

**Affiliations:** Josiah Macy Jr. Foundation, 44 East 64th Street, New York, NY USA; University College London, Gower St, London, WC1E 6BT United Kingdom; University of Illinois College of Medicine, 1853 West Polk Street, Chicago, IL 60612 USA; Department of Psychiatry and Behavioral Neuroscience University of Chicago Medicine, 5841 S. Maryland Ave., MC 3077, Rm M344A, Chicago, Illinois 60637 USA; Faculty of Health Sciences, Ben-Gurion University of the Negev, Beer Sheva, 8410501 Israel; University College London, Gower St, London, WC1E 6BT United Kingdom; Faculty of Medicine Hebrew University Hadassah School of Medicine, Braun School of Public Health and Community Medicine, Ein Kerem Campus, Jerusalem, Israel; Division of Otolaryngology Department of Surgery, Brigham and Women’s Hospital, 45 Francis St, Boston, MA 02115 USA

**Keywords:** Medical education, Physician workforce planning, Health professions workforce planning, Financing medical education

## Abstract

A 2014 external review of medical schools in Israel identified several issues of importance to the nation’s health. This paper focuses on three inter-related policy-relevant topics: planning the physician and healthcare workforce to meet the needs of Israel’s population in the 21^st^ century; enhancing the coordination and efficiency of medical education across the continuum of education and training; and the financing of medical education. All three involve both education and health care delivery.

The physician workforce is aging and will need to be replenished. Several physician specialties have been in short supply, and some are being addressed through incentive programs. Israel’s needs for primary care clinicians are increasing due to growth and aging of the population and to the increasing prevalence of chronic conditions at all ages. Attention to the structure and content of both undergraduate and graduate medical education and to aligning incentives will be required to address current and projected workforce shortage areas. Effective workforce planning depends upon data that can inform the development of appropriate policies and on recognition of the time lag between developing such policies and seeing the results of their implementation.

The preclinical and clinical phases of Israeli undergraduate medical education (medical school), the mandatory rotating internship (*stáge*), and graduate medical education (residency) are conducted as separate “silos” and not well coordinated. The content of basic science education should be relevant to clinical medicine and research. It should stimulate inquiry, scholarship, and lifelong learning. Clinical exposures should begin early and be as hands-on as possible. Medical students and residents should acquire specific competencies. With an increasing shift of medical care from hospitals to ambulatory settings, development of ambulatory teachers and learning environments is increasingly important. Objectives such as these will require development of new policies.

Undergraduate medical education (UME) in Israel is financed primarily through universities, and they receive funds through VATAT, an education-related entity. The integration of basic science and clinical education, development of earlier, more hands-on clinical experiences, and increased ambulatory and community-based medical education will demand new funding and operating partnerships between the universities and the health care delivery system. Additional financing policies will be needed to ensure the appropriate infrastructure and support for both educators and learners.

If Israel develops collaborations between various government agencies such as the Ministries of Education, Health, and Finance, the universities, hospitals, and the sick funds (HMOs), it should be able to address successfully the challenges of the 21st century for the health professions and meet its population’s needs.

## Introduction

The principal objective of medical education throughout the world is preparation of a competent workforce of highly skilled physicians who are prepared to handle the challenges they will encounter daily and are capable of responding to the changes in medical practice that inevitably will arise during their working careers.

The capacity of any nation to meet the health care needs of its population depends upon the specific policies and infrastructure that govern the education, postgraduate training, and support of its health care workforce, including physicians and other health care professionals. Since it takes at least 10 – 14 years to educate and train physicians, depending upon their specialty, development of a suitable national physician workforce requires a lot of forward thinking and coordination of resources. Unfortunately, most national governments manage education, health care, and other social services necessary to promote health through a variety of different agencies or ministries without any overarching coordinated workforce strategy or plan. Israel is no exception.

The authors of this paper were members of an *ad hoc* external committee appointed by the Israeli Council for Higher Education (CHE; Hebrew acronym = MALAG) to perform a review in 2014 of Israel’s four accredited medical schools. The committee’s General Report to the CHE has many recommendations related to a wide spectrum of school-specific, education-specific, and broad national policy issues.^1^

The committee, in writing this paper, recognizes that the Israeli health care system and medical education system have developed against a background of warfare and generations of high immigration. This has resulted in Israel’s having a very large number of physicians coming from varied medical educational backgrounds, a large increase in the population to be served by the health system, and great diversity of the population. The committee members were struck by the fact that Israeli medicine had so rapidly reached an advanced stage, and that there were many similarities between the critical issues faced in Israel and those in the United Kingdom (U.K.) and the United States (U.S.), countries with much longer histories of development of their health care and education systems.

In the U.K., U.S., and Israel, there are issues involving the development and financing of the physician workforce. Three key issues are: workforce planning; enhancing the coordination and efficiency of medical education across the continuum of education and training; and the continual need to examine the financing of all the stages of medical education and training so that they best support development and deployment of the needed workforce. In Israel, these issues extend well beyond the purview of the CHE, the agency that commissioned our review of medical schools and formal reports. The collaborative solutions necessary to achieve more efficient and effective production of the Israeli physician workforce will need to involve a varied group of stakeholders including several government, public, and private entities. Given the small size of the country, the intimacy of the academic and practice environment, and the degree to which both the educational and health care systems are supported by government funding, the committee sees great opportunity to attain even better results in planning and developing Israel’s national physician workforce.

## Methodologic basis of this report

In 2014, a committee of eight senior physicians, who have all had wide experience in medical education and collectively have had extensive experience in areas such as clinical care, research, and management, was assembled by the CHE to perform a periodic review of the four older, accredited medical schools in Israel: Ben-Gurion University, Hebrew University Hadassah, Tel Aviv University, and the Technion. Reviews of these schools had occurred in 2000 and 2007. This, however, was the first review committee whose chair was not Israeli and whose membership consisted primarily of non-Israeli physicians. The eight person committee included four from the U.S., two from the U.K., and two from Israel. Although not part of the original charge to the committee, four members also made a brief advisory visit to the new Bar-Ilan medical school in the Galilee. All school visits took place between mid-February and early June, 2014.

In preparation for the committee’s reviews, each of the medical schools produced an extensive self-evaluation report. The CHE specifies the format the schools are to use for the self-evaluation reports [1] and for the site visits. The required general categories of reporting are similar to those of the Liaison Committee on Medical Education (LCME) in the U.S. and cover the institutional setting, the teaching program (s) for an MD degree, admission of and services for students, faculty, and educational resources. The CHE also requires information on research, the self-evaluation process, and implementation of previous recommendations.

The LCME’s standards for accreditation and reaccreditation of medical schools in the U.S. are explicit [2]. There is a detailed data collection instrument for the review survey [3], and the format of the survey reports is specified in detail [4]. Ultimately it is possible for the LCME to evaluate whether a school has “met” or “not met” each standard, and those determinations guide accreditation and re-accreditation decisions and remedial actions. In the U.K., the body responsible for accreditation and review of medical schools, the General Medical Council (GMC), also has an explicit set of outcomes and standards for UME that guides reviews and decisions [5]. The CHE does have a 12 page general set of standards for medical education in Israel and a general format for the review committee’s report.^2^ The CHE format for the school’s self-evaluation report, however, is more loosely structured than the analogous LCME and GMC reports, particularly in expectations for the schools’ data collection and reporting. Moreover the CHE does not specifically reaccredit a medical school, but reviews and votes upon the reports of the *ad hoc* committee. Accepted reports with their recommendations then serve as the basis for follow-up with the schools.

The members of the committee read the self-evaluation reports in advance of their meetings and then spent approximately one work week on each school’s review, including a day of preparation, a three-day site visit, and a day developing an initial report including observations and recommendations. The site visits consisted of meetings with administration, faculty, and students with each school being allowed by the CHE to decide who the committee should meet within each category and what it should see. Additional writing and editing of the reports for each school was done remotely. The reports were submitted to the CHE which, in turn, distributed them to each school for reaction and response before they were presented to the Council.

The committee, informed by the reviews of each school and by the extensive prior experience of each committee member, also identified a large number of general, or national, issues that comprised the basis for the committee’s General Report to the CHE. The general issues included: the organization and conduct of the medical study programs across the country; institutional policies and practices among the medical schools’ parent universities; the health system in Israel and its interaction with the process of medical education; and a variety of other considerations of national importance.

This paper, written for a policy-making audience, has a section devoted to each of the three key issues mentioned above: workforce planning; enhancing the coordination and efficiency of medical education across the continuum of education and training; and financing medical education. Each section opens by referring to a table listing pertinent knowledge and observations of the committee.

## Issues and commentary

### Workforce planning

National planning and management of the physician workforce is “an important subset of overall health workforce planning and management, which contributes to a country’s having an effective and efficient health care system.” Nonetheless, it is “a multifaceted, difficult, and even controversial activity” [6]. Table 1 lists a number of the committee’s findings and observations that relate to workforce planning.

**Table 1:** Observations and Knowledge Related to Workforce Planning

• Israel knows it has shortages of physicians in certain specialties.
• Israel knows it has shortages of nurses.
• The OECD has stated that the strength of the Israeli health system is its primary care infrastructure, has predicted a growing shortage of primary care physicians, and has challenged the educational system to address this.
• Unlike the U.S., Israel does not have a cadre of nurse practitioners to help substitute for physicians.
• Medical schools are geographically distributed through the country.
• To address both a shortage of physicians and distribution of practitioners, there is a new medical school in the periphery.
• There now are financial incentives in Israel for physicians in certain specialties to train and practice in the periphery.
• The Israeli government has been urging all the medical schools to increase their class size.
• The four older medical schools seem to set their own priorities for the types of physicians they produce.
• Other than having developed plans to increase class size, a process that is well underway, none of the four older medical schools gave a clear indication it was contributing to a detailed national workforce plan.
• All Israeli medical schools have MD-PhD programs and emphasize their interest in producing physician scientists and in attracting more medical students into those programs.
o PhD’s, including MD-PhD’s are usually advised to take a postdoctoral fellowship abroad if they want to return to Israel in a faculty position.
o There appear to be no guarantees that there will be faculty positions for all who complete MD-PhD programs and do postdoctoral fellowships.

Planning a national workforce for the future requires having good information on the current composition of the physician workforce and its dynamics, e.g., age distribution, regional distribution, entrants to the profession each year, emigration of physicians from the country, retirements, etc. It requires knowing how adequately the existing physician workforce meets current population needs; and it requires projection of future population needs. The process also requires an understanding of how the workforce might be supplied and whether there are alternatives to physician services.

In the U.K. the GMC maintains a register that records specialty, including primary care. Recently the GMC has introduced a re-registration process termed “revalidation”.^3^ Although the impact of this on planning of the future U.K. health care workforce is as yet unknown, the committee believes that the absence of physician re-registration information in Israel impedes its having as good information about its current physician workforce and activity as it would need, and could have, for a more robust workforce planning process.

Depending upon the way it crafts its policies, a country can vary the degree to which it educates its own physicians or imports them after they have studied medicine in other countries. In the U.S., U.K., and Israel, a significant percentage of practicing physicians have gone to medical school abroad. In the U.S. and U.K., approximately 25 % of practicing physicians have received their medical degree from schools in other countries. All physicians in the U.S., irrespective of where they went to medical school must pass the U.S. Medical Licensing Examination (USMLE) in order to apply for medical licenses. In addition, in order to apply for a medical license in the U.S., all international medical graduates must complete graduate medical education (residency) within the U.S., even if they already have undertaken graduate medical education in another country. In the U.S. approximately 20 % of international medical graduates, or five % of all U.S. practicing physicians, are Americans who studied abroad.

In Israel, data on newly licensed physicians show that in the years since 1995, over 50 % each year have studied medicine abroad [7]. But, whereas in the 1990s most of Israel’s newly licensed international medical graduates were immigrants, now they are predominantly Israelis. In 2013, of 960 newly licensed physicians for whom country of origin and country of their UME were known, 14 % were immigrants, 43 % were Israelis who studied abroad, and only 43 % were graduates of Israeli medical schools. That was despite the fact that the number who were graduates of Israeli medical schools had already increased from approximately 300 per year in the years prior to 2008 to 400 per year by 2012 and 2013.

In the U.K., where most physicians and other health care workers either are employed by the NHS (National Health Service), or receive most of their revenue from it, there have been attempts at central planning of the physician workforce. In 2012, the Higher Education Funding Council for England (HEFCE) and the U.K. Department of Health published a review of medical and dental school intakes that included a review of what had happened following the third report of the Medical Workforce Standing Advisory Committee in 1997, and on this basis made recommendations for the next several years [8, 9]. In contrast to the U.S., the U.K. has a significantly larger primary care physician supply. Indeed general practitioners (GP’s) are the backbone of the British NHS and in recent years have been well compensated.

At the present time, both Israel and the U.S. are attempting to increase the number of physicians who receive a medical degree from schools within the country. In the U.S., although there were no new medical schools between 1986 and 2001, as a result of a projected physician shortage, and without a government mandate, 17 new schools granting the MD degree have opened since 2002. Combined with increases in class size in some older medical schools, overall enrollment is projected to increase by 30 % from 2002 to 2017 [10]. The U.S. now has 141 accredited medical (MD-granting) schools and 30 accredited osteopathic medical schools. There are 38 schools in various stages of development.

In the U.K., several new medical schools and programs have opened in recent years including some that offer post-baccalaureate four-year programs. There are now 33 medical schools in the U.K. including nine that have opened since 2000.

In Israel, the government requested substantial increases in the size of the undergraduate student body in the medical schools and has fostered the development of the new Bar-Ilan medical school [11]. As noted above, the previously existing schools have responded to the government’s request, and there has been an increase in the number of newly licensed graduates from Israeli medical schools [7]. Further increases are desired by the government and are planned by the schools. However, to handle the increased numbers of students, each of the schools has been concerned about the availability of clinical facilities for teaching, especially in hospitals. The schools also are concerned about having sufficient funds to meet a variety of needs associated with having larger numbers of students.

Across all three countries, despite increased numbers of medical students in existing and new schools, there are short supplies of various medical and surgical specialists, e.g., psychiatry and neurology in the U.S., emergency medicine in the U.K., and fields such as anesthesia, neonatology, and intensive care in Israel. In all three countries, there are growing shortages of primary care physicians, and we shall focus on that issue here.

The U.S. is known to have a higher ratio of specialists to primary care physicians than other developed countries and is widely believed to have a shortage of primary care physicians. Yet, data from the U.S. demonstrate that a locally higher primary care supply and a locally lower ratio of specialists to primary care physicians are associated with lower age-adjusted mortality and mortality from specific causes such as heart disease and cancer [12]. This has been a driver of efforts to increase the primary care supply.

The 2012 OECD report on health care quality in Israel [13] credited Israel “for shaping a strong primary health care system.” It said, “At a time when all OECD countries are grappling with more patients living with a chronic disease, Israel’s organization of primary health care services is geared towards supporting people who will live longer with more frequent health concerns”. It went on to state: “Israel’s ability to deliver health outcomes that are amongst the best in the OECD, despite spending less on health than most OECD countries, is attributable not only to a younger and healthier population, but also to the strengths of its primary care system.”

The OECD report also noted a paradox - that one of the major factors helping to ensure the adequacy of the Israeli primary care workforce in recent decades is now a factor placing the future physician supply in jeopardy, particularly in the periphery. Many physicians in Israel, including many of the family physicians, entered in the wave of immigration from the former Soviet Union in the early 1990s and many are now beginning to retire. The entire physician workforce in Israel has been aging [14], and this is particularly the case in primary care [15]. In short, Israel, with an increasing prevalence of persons who have chronic conditions [16] as well as a growing and aging population, needs to make a concerted effort just to maintain, not to mention enhance, its primary care workforce [13, 15]. It will have to address the fact that a significant number of the immigrant physicians arrived after medical school. Accordingly, their replacement is likely to require further expansion and refocusing of undergraduate medical education (UME) and graduate medical education (GME) resources.

The external committee saw very little emphasis on undergraduate ambulatory and community-based medical education among the schools it visited, with the exception of Ben-Gurion University. Even there, as at the other medical schools, exposure to family medicine consists of a several week block in the sixth year, which is late for influencing specialty choices. In a survey of sixth-year students in Israeli schools at Hebrew University Hadassah School of Medicine and Ben-Gurion University, only 25 % felt that family medicine was an “interesting and challenging specialty”; and this was lower than for specialties currently considered to be in short supply in Israel, e.g., anesthesiology (43 %) and general surgery (62 %) [17].

Although review of the mandatory rotating internship (*stáge*) was not part of the official charge to the committee, our understanding is that it allows the option of electing, but does not mandate, a family medicine rotation. In 2011, Afek et al. recommended significant changes in graduate medical education [18]. They observed, “The practice of medicine has changed in the last decade. Physicians no longer work largely solo but are part of interdisciplinary medical teams, and patient cases are more complex, as they suffer from multiple diseases. … There is also a shortage of physicians in Israel, as the number of new immigrant physicians has decreased over the last decade. Other medical professions and infrastructures are also lacking, including nurses, acute care hospital beds, intensive care beds and more.” To address these issues, they proposed “cancelling the internship” and dividing the residency program into two stages: “Two years in general medicine, surgery, or pediatrics, and the second part in subspecialties such as pediatric surgery, neurology, gastroenterology, cardiology and others. An option to continue residency in general medicine, pediatrics, or surgery will also be possible and must be encouraged.” If family medicine is added to the choices along with general medicine, general pediatrics, and general surgery, then restructuring GME along these lines could possibly be one way of increasing the supply of physicians in generalist fields.

The supply of all physicians, specialists and primary care, is relatively less in the periphery of Israel than in the center of the country [19], and the supply of specialists in the periphery has been even relatively lower than the supply of primary care physicians. There now are financial incentives to encourage taking residencies in the periphery and to practice in distressed specialty fields; but family medicine, despite the OECD Report, is not yet characterized as being a distressed specialty.

Any discussion of national workforce planning issues and primary care workforce supply should consider the potential contributions of non-physician clinicians. In the U.S. in 2010, there were approximately 209,000 practicing primary care physicians [20] out of a total of 850,000 licensed physicians [21]. In addition, there were 56,000 practicing primary care nurse practitioners and 30,000 practicing primary care physician assistants [22]. These non-physician clinicians add significantly to the supply of primary care services and mitigate the U.S. shortage of primary care physicians. Also, in the U.S., nurse practitioners, nurse anesthetists, other specialized nurses, and physician assistants take on roles that used to be performed primarily or exclusively by physicians. We do understand that Israel has a severe shortage of nurses, and only very recently, in the face of union opposition, has begun to develop advanced practice nursing.

In the U.S., the Patient Protection and Affordable Care Act (2010) includes several provisions that are expected to increase the supply of primary care physicians. These include increased reimbursement for primary care services, new slots for primary care residents, and expanded low-interest student loan programs for persons choosing a primary care field [23]. In the U.K. where there is currently a 9 % shortfall in the annual number of primary care physicians needed to maintain the present service, there is concern about recruitment of new primary care physicians. A task force established by Health Education England, a semi-independent governmental advisory body, and the Department of Health has made recommendations for achieving the needed number [24].

Thus, the current and growing primary care workforce supply shortage in Israel is not unique; but it does raise several national policy issues that directly or indirectly affect medical study programs. Though no one thing is in itself sufficient to solve the problem, there are several possibilities for making primary care more attractive and for enhancing the supply of primary care services. Undergraduate medical students need to encounter the subject early in their education; and they need exposure to enthusiastic role models, particularly ones who have been developed to be excellent teachers. In general, there needs to be increased emphasis on ambulatory and community-based medical education including during the internship (*stáge*) year. Financial incentives play a role, but there is also a need to invest in supportive infrastructure. In parallel, there should be an assessment of national primary care needs and the degree to which they are being met by physicians or can be met by other health professionals.

A comprehensive approach to developing the workforce of health professionals that Israel needs for the future and addressing existing and looming shortages will require the interaction and collaboration of multiple stakeholders, including government ministries and agencies. There will need to be clarity at the highest levels of government about who has the lead for the key pieces such as understanding the supply of, and demand for, the health professional workforce, setting policies that support development and retention of health professionals, and aligning the financing of health professions education and health care delivery.

### Enhancing the coordination and efficiency of medical education across the continuum of physician education and training

It is important to consider how best to coordinate the several phases of education and training of a physician to meet workforce needs as effectively and efficiently as possible. The series of phases is often referred to as the continuum, trajectory, or arc of medical education. Relevant committee findings and observations are shown in Table 2.

**Table 2:** Observations and Knowledge Related to Enhancing the Coordination and Efficiency of Medical Education across the Continuum of Physician Education and Training

• Successful applicants to medical schools in Israel must have top scores on the matriculation examinations given to all high school students.
• In addition, all medical schools in Israel also base their selection on an assessment of the applicant’s humanistic qualities.
• The standard education of Israeli physicians consists of: three preclinical years; three clinical years; a one-year rotating internship (*stáge*) that must be completed before the MD degree is granted; and four or more years of residency, depending upon the specialty.
o There also are relatively new four-year, undergraduate medical teaching programs for persons who already have a bachelors or advanced degree in the sciences.
• The course of study and training for an Israeli student who chooses to go into a primary care field such as family medicine or pediatrics, is at least as long as in the U.S.
o By comparison, the U.S. student who has four years of college, has about three of those to pursue academic interests other than those required for medical school admission; whereas, the curriculum for the Israeli student is fully prescribed.
• In most Israeli schools there has been little integration of the basic sciences and clinical knowledge.
o Students voiced strong complaints about the lack of relevance of what they were taught in the basic sciences to their future careers.
• The majority of undergraduate teaching, especially in the preclinical basic science curricula, is lecture-based.
o On average, attendance at lectures is poor.
o Faculty members report that Israeli medical students want to be “spoon-fed.” Students report that they would prefer more interactive teaching.
• Almost all evaluation is done by multiple-choice question (MCQ) examinations.
o Faculty report that given the numbers of students they must evaluate, they have no alternative to MCQs.
• Responsibility for the continuum/trajectory of physician education is divided.
o Responsibility for undergraduate curricula rests within the universities.
o Responsibility for the rotating internship (*stáge*) rests with the collective group of medical school deans, the Deans Forum, and the Israel Medical Association.
o Responsibility for residency programs rests with the Israel Medical Association.
• The CHE performs a periodic external review of the undergraduate teaching programs.
o This is not coordinated or integrated with review of the rotating internship (*stáge*), or review of graduate medical education programs.
• Israeli medical schools do not have an explicit set of competencies to guide curriculum development.
• Individual courses and clerkships in Israeli medical schools generally do not have specific learning objectives to form the basis for student and faculty accountability.
• The majority of clinical education in Israel has been in hospital settings.
o Increasingly health care delivery is occurring in ambulatory settings.
o All medical schools report scarcity of hospital resources for teaching, especially as class sizes are increasing in response to government requests.
o Ambulatory medical education is occurring increasingly in the U.S. and U.K.
o Ambulatory education requires facilities suitable for teaching and learning, faculty development, and appropriate incentives to engage the faculty.
o The committee did observe teaching of undergraduate medical students in two clinics jointly developed by Clalit and Ben-Gurion University.
• Interprofessional education (IPE) is important for preparing learners to practice effectively in teams.
o IPE is occurring in most schools in the U.S. and U.K.
o IPE is occurring at only one university in Israel.
• Promotions for clinical faculty are generally based on research criteria similar to those for pre-clinical faculty.
o Teaching ability, though considered in promotions, is not a deciding criterion.
o Many students have a job while in medical school; and it is common for students to leave their clinical clerkships in mid-afternoon in order to work.

**Table 3:** Observations and Knowledge Related to Financing of Medical Education

• The government of Israel supports higher education, including medical education and other health professions education, through a separate entity called VATAT.
• Funding goes directly to universities and colleges. Even though it is based upon the educational programs of those institutions, the distribution of it within the institutions is a local responsibility.
• In the first decade of the 21^st^ century there was a cutback in funding
o In the second decade, there has been restoration of some of the funds.
o Each medical school expressed concern about the number of faculty positions for which it had funding, particularly basic science faculty.
• Three Israeli medical schools have four-year English language programs for non-Israelis with prior baccalaureate degrees that have high tuitions.
o These programs share faculty with the Hebrew language programs.
o English-language programs produce significant revenue for the university.
• Schools have understandable concerns that development of ambulatory medical education will require significant financial resources that do not currently exist.
• Promotions for clinical faculty are generally based on research criteria (see Table 2).
o The majority of clinical teachers do not have any university appointment.
• Academic promotions to senior faculty positions in Israeli universities carry financial benefits including supported sabbatical time and supported meeting travel.
o Most clinical teachers work for the health system.
o Universities are expected to fund the benefits associated with academic promotions.
• Students reported needing to work while in medical school to gain necessary income to support family obligations or the high cost of living in some areas.
• The support for the PhD component of MD-PhD programs tends to be short (2–4 years).
o It is difficult to take on an important and challenging research project when supported research time is short.

Over 100 years ago, the Carnegie Foundation for the Advancement of Teaching published a book-length report by Abraham Flexner on medical education in the United States and Canada. It is known widely as “The Flexner Report” [25]; and it changed the nature of medical education in the U.S., Canada, and many other countries. It standardized the format into pre-clinical and clinical years and placed an emphasis on scientific knowledge as a basis for modern medicine and medical education. The idea that medical education should be grounded in relevant science and evidence is now taken as a given. But, over the years, it has become apparent that what was regarded as science at that time – biological and physical science - is a necessary but not sufficient basis for developing effective physicians.

In 2010, in commemoration of the 100^th^ anniversary of the Flexner Report, the Carnegie Foundation published a book outlining the need for and steps to achieve “the next level of excellence” in medical education [26]. Importantly, the scope of the book includes not just medical school, or UME, but also residency, or GME. The authors of the 2010 report wrote, “There is a need to motivate continuous learning and improvement across the whole arc of medical training. Those who teach medical students and residents must choose whether to continue in the direction established over a hundred years ago or take a fundamentally different course, guided by contemporary innovation and new understanding about how people learn.” The recommendations of the 2010 report do not reject the need for a strong science base, but rather are built on an understanding of the shortcomings of 20^th^ century medical education efforts in an environment that is changing and expanding.

In the past, the education of medical students in basic science was mostly detached from its clinical applicability. Basic science courses were taught as separate subjects by highly accomplished, but clinically unsophisticated scientists; and learning in the clinical settings was often not informed by rapidly advancing knowledge in the basic sciences. Underrepresented in the curriculum were relevant aspects of social sciences - such as psychology, sociology and anthropology; statistical analysis and population-based thinking; and communication, human interrelationships, and principles of team management. Clinical experiences used to occur after the completion of the science courses and took place almost exclusively in hospitals; but today, more and more clinical care is being delivered in ambulatory settings including the patient’s home. The past also emphasized acquiring, usually by rote learning, a large body of information on which the physician could depend. Today, with recognition that the rate of development of new knowledge keeps growing rapidly and with new tools such as the internet that can provide the latest information, it is even more essential than in the past that learners be prepared to acquire the knowledge they need, as they need it.

The 2010 Carnegie report took into account existing efforts to address these issues and built upon them. Its recommendations, addressed primarily to the U.S., include:

- Standardize learning outcomes and assess competencies over time.
- Strengthen connections between formal and experiential knowledge across the continuum of medical education.
- Incorporate more clinical experiences earlier in medical school.
- Provide more opportunities for knowledge-building later in medical school and throughout residency.
- Promote learners' ability to work collaboratively with other health professionals, such as medical assistants, nurses, pharmacists, physical therapists and social workers.
- Support learners' responsibility for quality of care, team performance and their own learning while providing skilled supervision.
- Make professional formation an explicit area of focus in medical education through strategies such as formal instruction in ethics and reflective practice, exploration of the role of the physician-citizen and establishment of more supportive learning environments.
- Cultivate a spirit of inquiry and improvement in learners and in health care teams; this spirit supports both innovations in daily practice that translate into better service to patients, system improvements and improved patient outcomes as well as the development of larger research agendas, new discoveries, and knowledge building.
- Be more intentional about selection, development and support of teachers and medical educators.

In its review, our committee observed that the original Flexnerian model had been incorporated into Israeli medical education, but the recommendations of the new 2010 Carnegie Report that are being adopted in the U.S. and U.K. had yet to be introduced.

The external committee found that in most of the Israeli schools there remains a sharp separation between pre-clinical and clinical education. These two components of UME are regarded as separate “silos”. The committee learned that the schools, despite some specific positive efforts, generally have not organized their preclinical education to establish clearly that the material being taught in the basic sciences is relevant to clinical medicine. Furthermore, at some of the schools, basic scientists with junior faculty appointments reported being called on to teach subjects that they do not feel qualified to teach.

The predominant methods used for pre-clinical teaching in Israel do not promote engagement of medical students. Faculty consider lectures to be an efficient way for a limited number of teachers to handle a large and increasing number of students; and almost all the preclinical teaching consists of frontal lectures. In a few instances, case studies and problem-solving were being incorporated into courses. This approach was associated with both student and educator enthusiasm. It was, however, the exception.

In other countries, such as the U.S. and U.K., an increasing percentage of preclinical studies have been, or are being, converted to active learning vs. traditional lectures. Nara, et al., who visited 35 medical schools in 12 countries around the world, addressed this subject in a 2011 report [27]: “Formally, the knowledge of medicine has been taught by teachers through lectures in a large theater. Students have learned mainly medical practice by observation at the outpatients’ clinic and ward. Although these education methods play important roles even at present, most medical schools have recently introduced new education methods to promote clinical training for medical students. Students get a large amount of recent medical knowledge by tutorial system such as problem-based learning and team-based learning. For this purpose, an e-learning system has been developed in most medical schools.”

Nara and his colleagues also observed that “Integrated courses [combining] basic medicine and clinical medicine have been introduced in many medical schools in the world. For example, students simultaneously learn basic bacteriology and clinical infectious disease at the same lecture or tutorial.” In the U.S. and U.K. there are some very interesting efforts to introduce learning experiences in clinical settings early in the course of study [28] and to evaluate the effects of early clinical experiences [29].

In their site visits to medical schools around the world, Nara et al. noted that clinical education involves having “students belong to the medical team as staff and do medical practice under the supervision of attendants.” They further observed that “this clinical clerkship system is most advanced in the U.S. and Canada [27].”

The committee’s understanding was that in Israel meaningful clinical experiences and clinical engagement generally began late in UME. There appeared to be many more small group lecture sessions in the clinical clerkships and relatively fewer direct clinical experiences than in the U.S. and U.K. While some of the part-time work that many Israeli students engage in can provide clinical experiences, such jobs are not uniformly available. The jobs that are available are not routinely and purposefully integrated with their curriculum and monitored appropriately. Furthermore, in the clinical years, we were told that it was common for Israeli students to leave their organized clinical placements in mid-afternoon for their jobs. This limits the degree that students could participate in the continuing clinical care of patients and be considered, as students are in the U.S., members of the clinical team caring for a specific set of patients.

It is very important to note the emphasis throughout the 2010 Carnegie Report recommendations on the entire continuum, arc, or trajectory of medical education from selection and matriculation of students to development of competent and professional physicians. This emphasis is aimed at developing a suitable cadre of physicians more effectively and efficiently. To do so, it is essential to coordinate across the settings of medical education and clinical care, to build competence in a stepwise fashion, and to ensure that essential competencies such as professionalism and capacity for lifelong learning are emphasized at all times in all settings.

In other countries there is a growing emphasis on ambulatory and community-based medical education. In Israel, the enlarging undergraduate class sizes in response to government requests have led to great concerns by the medical schools about limited clinical resources for teaching. Certainly there are limited hospital resources, but Israeli medical schools appear to over-emphasize the need for hospital-based clinical education and under-emphasize ambulatory and community-based education.

Our findings raise several policy-relevant issues:

Even though teaching is a requirement for appointments and promotions in Israeli faculties of medicine, the primary criterion is scholarship as defined by publications in scientific and medical journals, not by teaching or clinical program innovations. This contrasts with a trend to broaden the definition of scholarship in U.S. medical schools. Even highly reputed research universities such as Harvard University and the University of Chicago have developed a more inclusive definition of scholarship.^4^ At Harvard, an area of excellence is chosen by each faculty member. Those who seek promotion in the teaching and educational leadership area must demonstrate a progressively broader reputation for leadership – ranging from local to regional to national and international – as well as scholarship. Scholarship may include: “publication of original research, reviews, and chapters; educational material in print or other media such as syllabi, curricula, web-based training modules and courses; and/or, educational methods, policy statements, and assessment tools developed”.

 Although there have been resemblances between the situations in the U.K. and Israel, the U.K. situation is evolving. In the U.K., teaching and education domains have been introduced recently into the regulatory framework related to promotions, and can be incorporated into the documentation required for re-registration.^5^ Tackling the question of how to handle and reconcile tensions between clinical service needs, education and training requirements, and innovation and research, remains an issue that is critical to the future of academic medicine [30]. If Israeli clinical teachers had appropriate incentives to assume teaching responsibilities not only in clinical settings but also in the earliest phases of medical education, it would then be possible to integrate better the current pre-clinical and clinical phases of education of Israeli physicians.

There is an opportunity to establish national policies and practices that could lead to more efficient and effective medical teaching using currently available technologies and approaches. For example, the committee felt that lectures on the same subjects need not be given separately at each of the medical schools or at each of the teaching hospitals. To the extent that oral didactic materials are needed, the most capable teachers could deliver polished lectures that could be recorded and accessed electronically by students at each school. The presentations could be updated periodically. A central resource could be established to do the recording, editing, and maintenance of such material. Online availability of uniformly excellent oral and written didactic material would enable teaching to occur in “flipped classroom” settings^6^ or other forms of active learning.^7^

An important reason for fostering more active learning in Israeli medical education is stimulating a sense of inquiry and problem solving. Both of these are essential for life-long learning. Lectures are not as effective as active learning methods in stimulating inquiry in the students [31]. To make the transition from lecturing to active learning, especially with large classes, there will need to be extensive faculty development in each of the schools. Central resources could be developed to support faculty development across the country. The new national organization for persons interested in medical education could facilitate improvements in educational practices and scholarship if it is appropriately supported.^8^ This new central resource might also be useful in developing a national set of competencies that could guide curriculum development in each of the medical schools as is now occurring in the U.S. [32].

Promoting active learning will not only enable physicians to benefit their patients as new knowledge is accumulated during their years of practice, but also may stimulate more Israeli medical students, residents, and physicians to engage in all forms of research and inquiry. Lectures have been shown not to be the most effective way to foster a spirit of inquiry, and they are out-of-step with traditional graduate study, with its emphasis on individual inquiry and small-group seminars. Each medical school stated the desire to develop more MD-PhDs. That is more likely to occur if the entire educational process of medical students stimulates more inquiry. Similarly, medical students are more likely to develop lifelong learning skills if their teaching program, starting at the earliest stage, consistently stimulates inquiry.

Simulation provides an opportunity for learners to develop competence in both cognitive and technical skills, and the ability to work in teams in an environment that is safe for them and for patients. In the U.S., in pre-licensure nursing education, a ten-center collaborative study has shown that simulation can replace as much as 25–50 % of clinical hours [33]. Indeed, simulation centers are nearly universal in U.S. and U.K. academic medical centers; and almost all medical students in the U.S. have multiple experiences involving simulation [34]. The Israel Center for Medical Simulation (MSR) based at Sheba Hospital is internationally recognized for its excellence. It has programs for physicians including the national mandatory interns' workshop, and the national board examinations in anesthesia and emergency medicine. Its activities provide a model for having a centralized UME resource. MSR and the Simultech Center at Meir Hospital are used to some extent in medical student teaching and faculty development at Tel Aviv University, but otherwise there is little utilization of clinical simulation in UME in Israel.

The development of ambulatory and community-based teaching should be central to coordination across the trajectory of medical education. This partly involves creating appropriate incentives for faculty in ambulatory and community-based settings and faculty development programs. It also requires developing the physical facilities so that they are appropriate for both efficient clinical care and effective teaching. The committee did observe teaching of undergraduate medical students in two clinics that have been developed jointly by Clalit Health Services and Ben-Gurion University; and these clinics demonstrate the possibility of collaborative development of ambulatory medical educational facilities in Israel.

Yet another issue is whether Israel can move from time-based to competence-based curricula. Currently, students entering medicine spend three preclinical years, three clinical years, one internship year, and at least four years of residency depending upon the specialty. This is a time-based curriculum. In contrast, a competence-based curriculum would specify levels of competence, or milestones, for each of several competencies that a physician was expected to gain but allow the time in which the learner acquired the competencies to vary [35, 36]. A competence-based approach opens new possibilities since learners will acquire basic competencies or reach successively higher levels of competence at different rates. At least in theory, the time a learner spends in each phase of the education and training process can be variable – longer or shorter than the currently allotted time. Israel already allows flexibility in the start date for internship (*stáge*) that may facilitate its moving to a competence-based curriculum in which the time for UME varies with the learner’s ability to acquire necessary competencies. Also, with a competence-based curriculum it becomes possible to guarantee to the population that each physician who emerges from a training program meets high standards of competence. And, even if the duration of the study program remains fixed, a competence-based curriculum should permit learners who reach acceptable levels of a competence before the allotted time is up either to acquire mastery in that area or to work on acquiring additional areas of competence.

The external committee was asked to look only at UME. In the U.K., the importance of the continuum of development of a physician from entry as an undergraduate through internship and residency into the career-long phase of continuing professional development has been recognized very recently in a decision that the GMC, a regulatory body, should be responsible for evaluating and monitoring all the phases [37]. The external committee believes that a national review from such a perspective in Israel could provide valuable lessons and lead to more effective and efficient development and maintenance of a competent physician workforce. The individual components of the review need not all be the responsibility of one committee; but oversight of the review components and their coordination could be handled by one group. If the individual component reviews were highly structured, the coordinating group could synthesize them readily.

### Financing of medical education

Financing of medical education generally involves a number of policy considerations and decisions. These relate in part to whether medical education is considered a public good; in part to the fact that the various stages in the education and training of a physician that need coordination might have different financing mechanisms; and in part to the fact that the education and professional preparation of physicians and other health professionals occurs in clinical settings as well as in traditional educational institutions. This contrasts with the education of most other professionals, scientists, and scholars. Not surprisingly, different countries take different approaches to these issues.

In the U.S., UME occurs in several types of medical schools including private medical schools with high tuitions, state medical schools with somewhat lower tuitions, and one military medical school, the Uniformed Services University of the Health Sciences, where tuition is waived in consideration for several years’ military service. Students in all civilian medical schools are eligible for a variety of loans and some need-based scholarships. A large percentage of graduating medical students have acquired a significant amount of debt due to loans during both their college and medical school years [38]. During the GME years, residents receive a salary. It is lower than it might be if one were simply paying for the medical services delivered by the residents since there is not just service but also an educational component. A significant amount of the funding to academic health centers (AHCs) to support their residency programs comes from the U.S. federal government through the Medicare program. The complex and controversial financing of GME through the national Medicare program that is the major payer for the health care of older Americans raises difficult questions about the specific residency programs supported, the adequacy of support, and the sources of support [39, 40].

Higher education funding in both the U.K. and Israel derives primarily from two sources: a central funding organization that gets its money from the government; and student tuitions. In the U.K., HEFCE funds undergraduate medical and dental training jointly with the NHS. HEFCE support consists of a grant that is part of the annual funding allocations to each university. It is calculated on the basis of the number of medical and dental students in each medical and dental school; and those numbers are determined by a target for each school.^9^

In addition, students may be eligible initially for student loans and later for scholarships to help support tuition fees. In the first four years of the standard five-year UME curriculum, students can apply to a government organization, Student Finance England.^10^ From year five, tuition fees can be paid by the NHS Student Bursary Scheme, a scholarship program. Students can apply for a means-tested NHS scholarship “to cover maintenance costs and a reduced maintenance loan from Student Finance England.”^11^ There is also the possibility that students who apply will receive a small non-means-tested scholarship award.

The committee’s findings and observations with respect to financing of medical education in Israel are shown in Table 3. The principal funding stream for UME in Israel is determined by VATAT, the planning and budget committee of the CHE. VATAT is affiliated with the Ministry of Education; and it distributes the funds for higher education to the universities. In turn, the universities are then responsible for distributing the funds to their various faculties. This component of the overall financing of medical education in Israel at the level of the university is structurally similar to that in the U.K. However, as noted above, in the U.K. there is shared funding of the award to the university from the usual source of funding for higher education (HEFCE) and the NHS, the major funder and provider of health care delivery services.

Israeli universities do charge tuition, and the tuition has been rising. Annual tuition fees in Israeli medical schools (approximately 14,000 shekels = 3,650 dollars) are higher than in Europe (approximately 500 Euros = 560 dollars). In the U.K., annual tuition is higher than elsewhere in Europe (9,000 pounds = 14,100 dollars); but tuition in both Israel and the U.K. is much lower than in U.S. medical schools which average over 31,000 dollars at public universities and over 53,000 dollars at private universities. Students in the U.S. have school responsibilities that do not permit significant paid work; and they do accumulate very large levels of debt by the time they graduate medical school. In 2014, 84 % of graduating U.S. medical students had debts reflecting their college and medical school expenses; and the average indebtedness for those students with debt was 180,000 dollars [41]. Israel, however, unlike the U.S., many European countries, and the U.K., does not have significant student-loan programs [42]. Israeli students report that tuition charges and living expenses are major reasons for their needing to take part-time jobs during the course of UME despite financial support from their parents.

In Israel a university faculty appointment carries monetary benefits such as sabbaticals and travel funds. In consequence, a faculty appointment represents a financial commitment for the universities. The basic science faculty members are all employed by the universities and accordingly receive university appointments. Young faculty members are expected to do research and teaching. Assuming the university has chosen persons whose research is productive, those faculty members will be promoted within the university as their careers develop. In effect, all that has been built into the VATAT funding formula for the university. In contrast, the clinical teachers are employed by the health system, not generally by the university. Physicians, unlike PhDs, generally do not get salary support for doing research; but in order to be eligible for university appointments and promotions they still are expected by the universities to have done and published research. Thus, they have to do research during uncompensated time, often nights and weekends. And, were the universities to have a more inclusive definition of scholarship that allowed many more clinical teachers to be eligible for appointments and promotions (see above), then the universities would have to make a financial commitment to a group of people they currently do not support. The funds would need to come either from universities themselves or from funds allocated to the delivery system. In either case, there currently are no funds from either source; and the majority of clinical teachers in Israel do not have a university appointment. This is a disincentive to clinicians engaging in medical school teaching during the pre-clinical years or increasing their teaching commitments in the clinical years.

There are financial implications to providing more ambulatory-based clinical education. Teaching undergraduates and even residents in the early years of GME in ambulatory settings is known to reduce the clinical productivity of the faculty during the time when they are teaching. Only in the later years of GME do residents usually have the capability of contributing to overall clinical productivity. Thus, if clinicians working in the ambulatory sector who are being paid almost exclusively to be clinically productive are to become more involved in clinical teaching, there will need to be a source of financial support for that teaching. An increase in ambulatory and community-based teaching also involves the need for capital funds. It will require upgrading facilities so that there is space for learners and for related teaching activities. Both this capital expense and the benefits it could provide should be considered against the costs and benefits of expanding hospital facilities and hospital-based clinical teaching.

In light of VATAT’s current practice of only providing funding to each individual university, developing and sustaining central resources that can be shared by all the medical schools requires all of the educational institutions to agree jointly to equitable funding for each resource. An alternative would be to supplement the current arrangement with a central source of funding for shared resources and developing a process for planning and allocating funds to the highest priorities.

MD-PhD programs are important for developing physician researchers. Though there is some variation, in the majority of Israeli medical schools a student enrolled in an MD-PhD program can receive funding for doing research for only a relatively short period. It has been as short as two years, although three is more common, and sometimes it is possible to extend to four years. In the U.S., support for the research of MD-PhD students is generally four years and can be longer, particularly if the trainee and his/her mentor obtain grant support from one of several sources including pre-doctoral fellowships from the National Institutes of Health.^12^ The committee believes that an expectation of a shorter period of dedicated funding for research can lead to the PhD projects of MD-PhD students being less ambitious than they should be to develop a highly competent investigator and to projects that would be less ambitious than for other doctoral candidates. Also, in Israel, the dual degree student who cannot complete the work in the dedicated research time must continue to spend considerable time on his/her research while attempting to gain clinical experience. This is suboptimal for developing a highly competent MD and PhD. Addressing it would require having sufficient sources of funds for the PhD portion of MD-PhD programs.

Three medical schools (Ben-Gurion University, the Technion, and Tel Aviv University) have four-year English language medical study programs. These programs provide the universities with substantial tuition revenue that complements VATAT funding. With the government’s desire that the Hebrew-language medical school class sizes increase to accommodate national needs, the nation’s hospital resources for teaching are overburdened. Were the English language programs either to be reduced or eliminated to free up some of the hospital teaching resources, there would be a need for alternate funding sources to replace present tuition revenues. Hebrew University, though it does not have an English language program, appears to face a similar clinical resource strain. It has 60 students per year in the military medicine (Tzameret) program, funded by the military, and the smallest number of affiliated hospital beds per medical student [43].

An important first step for Israel would to understand that medical education raises the many financing issues above. A next step would be to establish a collaborative process to address them. It is likely that the process would involve several relevant government ministries and agencies, with input from educational institutions, the sick funds (HMOs), and health care delivery organizations. The distribution of funds for teaching between the universities and the clinical facilities with which they are affiliated seems to have been a contentious issue in the past [43], and will need to be addressed further going forward.

## Additional discussion and conclusions

We believe that Israel needs to reform its medical education system to meet the needs of its population for the 21st century. It will be essential to determine workforce needs and examine the capacity of Israel’s medical schools and health care delivery system to develop the number of physicians the country needs. They must be developed effectively and efficiently across the entire trajectory of education and training. Israel’s primary care workforce needs to be augmented in order to sustain the successes recently praised by the OECD [13]; and at the same time Israel must produce appropriate numbers and types of competent clinical specialists. Physician-researchers, physician-managers, and public health physicians are all necessary too, and they add value to the nation. Different universities may, due to their particular strengths, produce more of one type of physician than the others, e.g., MD-PhDs; but in a small country no university should be allowed to decide unilaterally to produce a preponderance of one type of physician. This follows both from an obligation to meet national needs and from the fact that the major source of funding is governmental.

Likely reforms within UME include: adopting interactive learning methods and competence-based education; providing earlier exposure to clinical medicine and more hands-on experiences; increasing significantly the use of ambulatory and community-based clinical facilities for teaching; and ensuring that all physicians will have excellent life-long learning skills to cope with the pace of change that is occurring in medicine and is expected to continue.

We definitely believe that Israel can develop an excellent and efficient mechanism for educating the physicians it needs. Israel has the advantage of having a small number of medical schools and a limited number of clinical facilities that will require coordination. It also has the potential advantage of government being the major source of funds both for education and clinical care. Yet, Israel will need to bring into balance the traditional values of higher education institutions and health care delivery organizations. Universities are legitimately concerned about academic freedom and the importance of scholarship. Clinical facilities are legitimately concerned about providing excellent care to their patients. Each type of institution also has legitimate concerns about financial solvency, a pre-requisite for achieving its major mission. Since both the universities and health care delivery system are currently feeling substantial financial pressures, ensuring that the sources of funds to ensure development of the workforce that Israel needs are adequate will require a collaborative effort involving the institutions and their current and potential funders.

We know that Israel was able to prepare an excellent cadre of physicians for the needs of the 20^th^ century. If Israel does develop collaborations between various government agencies such as the Ministries of Education, Health, and Finance, the universities, hospitals, and the sick funds (HMOs), it should be able to address successfully the challenges of the 21^st^ century for the health professions and meet its population’s needs.

## Footnotes

1 The external committee submitted its General Report to the CHE on August 19, 2014. It was discussed by a subcommittee of the Council on December 16, 2014, and accepted by the Council on February 10, 2015, marking a formal end of the *ad hoc* committee.

2 These documents were supplied to the committee, but are not posted on the CHE web site.

3 See: www.gmc-uk.org/doctors/revalidation.asp

4 As examples see the criteria for promotion at Harvard Medical School and the University of Chicago Pritzker School of Medicine: Harvard - facultypromotions.hms.harvard.edu/promotions.pdf; Chicago - https://webshare.uchicago.edu/users/vvv1/Public/Pathways_pdf.pdf

5 www.gmc-uk.org/doctors/revalidation.asp

6 “Flipped classroom is a form of blended learning in which students learn content online by watching video lectures, usually at home, and homework is done in class with teachers and students discussing questions and solving problems. Teacher interaction with students is more personalized - guidance instead of lecturing.” See: http://en.wikipedia.org/wiki/Flipped_classroom

7 There are a variety of sources of information on active learning. For instance, see: web.calstatela.edu/dept/chem/chem2/Active/ for definitions, techniques, and references on active learning.

8 The new organization, called HEALER, had its first national meeting in September, 2014.

9 See: http://www.hefce.ac.uk/lt/Healthcare/hefcerole/

10 See: https://www.gov.uk/student-finance/overview

11 See: www.nhscareers.nhs.uk/explore-by-career/doctors/training-to-become-a-doctor/undergraduate-medical-education/financial-support-for-students-on-degree-courses-in-medicine/

12 See: https://www.aamc.org/students/research/mdphd/financial_md-phd/
